# Assessment and management of neurogenic claudication associated with lumbar spinal stenosis in a UK primary care musculoskeletal service: a survey of current practice among physiotherapists

**DOI:** 10.1186/1471-2474-10-121

**Published:** 2009-10-01

**Authors:** Christine M Comer, Anthony C Redmond, Howard A Bird, Philip G Conaghan

**Affiliations:** 1 Section of Musculoskeletal Disease, Leeds Institute of Molecular Medicine, Section of Musculoskeletal Disease, University of Leeds, Chapel Allerton Hospital, Leeds, LS7 4SA, UK

## Abstract

**Background:**

Neurogenic claudication (NC) is the clinical syndrome commonly associated with lumbar spinal stenosis (LSS). Non-surgical management is recommended as initial treatment, but little is known about current practice in relation to the assessment and management of these patients in the non-surgical setting.

**Methods:**

We conducted a questionnaire survey of physiotherapists in a large UK primary care musculoskeletal service which provides a city-wide multidisciplinary assessment and treatment facility for patients with spinal and other musculoskeletal problems. Data on therapists' recognition and management of patients with NC and LSS were collected.

**Results:**

Fifty out of 54 therapists completed questionnaires, and all but one of these identified a clearly recognised posture-related clinical syndrome of NC. Almost all respondents (48: 96%) reported the routine use of physiotherapy treatments. In particular, advice and education (49: 98%) along with an exercise programme (47: 94%) incorporating flexion-based exercises (41: 82%) and trunk muscle stabilising exercises (35: 70%) were favoured.

**Conclusion:**

Musculoskeletal physiotherapy clinicians in this survey recognised a clear clinical syndrome of NC, based on the findings of posture-dependent symptoms. Most therapists reported the routine use of flexion-based exercise, reflecting recommendations in the literature which are based on theoretical benefits, but for which trial evidence is lacking. There is a need for research evidence to guide the choice of physiotherapy treatments.

## Background

Neurogenic claudication (NC) is described as the classic clinical presentation of lumbar spinal stenosis (LSS), a degenerative condition of the lumbar spine normally affecting adults over the age of 50 [1,2]. Despite being a common condition associated with substantial disability and healthcare costs [2-4], little is known about the current management of patients with NC, especially prior to surgical intervention.

Symptoms of NC are described as pain, paraesthesia or cramping of one or both legs, brought on when walking and relieved in sitting [5]. The effect of posture on symptoms is the primary distinguishing feature of NC: symptoms are typically exacerbated when the spine is extended (in upright stance when standing or walking) and eased when the spine is flexed (stooping forwards or sitting). Clinical symptoms are believed to result from stenotic changes (narrowing) exacerbated by posture-related compression causing neural and microvascular compromise of the cauda equina and lumbosacral nerve roots [5-9]

Not all patients with LSS have symptoms of NC, but an age of 65 or over, the presence of radiating leg symptoms aggravated by walking and relieved in sitting, and poor balance are common [10,11]. It has been reported that these findings have a high sensitivity for identifying patients with radiological stenosis but specificity is variable. In consequence, a recent review paper concludes that no firm conclusions on the diagnostic performance of clinical or radiological tests can be drawn [12].

Whilst the clinical syndrome of NC is usually associated with LSS, the pathoanatomic condition of LSS determined by MRI or CT imaging of the spine is not always symptomatic. Furthermore, there is a lack of agreement on the radiological measurements constituting stenosis. As the correlation between radiological findings and clinical presentation is poor [13], this pathoanatomical diagnosis of LSS may have little clinical relevance. The clinical syndrome of NC, on the other hand, provides a recognisable and meaningful subgroup of chronic low back pain patients who can be identified by the presence of posture-related clinical symptoms. Expensive spinal imaging may therefore be unnecessary except where surgical treatment is planned.

Surgery may not always be the treatment of choice for patients with NC related to LSS, except in cases presenting with severe and persistent pain and disability or where there are signs of progressive neurological deficit or cauda equina compression. Indeed, non-surgical interventions are almost universally recommended for initial treatment [2,14-16]. The few trials and case studies of LSS management suggest that exercise therapy consisting of flexion-based movements and lumbo-pelvic stabilisation exercises may be beneficial [17-23]. However, there is no agreement on which non-surgical treatments are most effective. Nor are there published data describing current practice in terms of clinical recognition and management of patients with NC in the non-surgical setting. This survey aimed to explore clinical recognition of NC, current patterns of patient assessment, and current management of these patients within a large, primary-care based musculoskeletal service.

## Methods

### Setting

The survey was carried out in the Leeds Primary Care Trust Musculoskeletal Service in the United Kingdom. This is a multidisciplinary interface/clinical assessment and treatment (CATS) type service providing an assessment and treatment facility for patients with non-surgical musculoskeletal conditions. Established in the year 2000, the service now receives around 40,000 referrals each year from GPs throughout Leeds. It is staffed by 60 physiotherapists, three musculoskeletal physicians, and four biomechanical podiatrists, supported by administrative staff. The service provides patient care in 40 local settings across the five regions of the Leeds Primary Care Trust. Extended Scope physiotherapy practitioners among the staff have the facility to refer patients to hospital consultants, including pain-management specialists, orthopaedic surgeons and neurosurgeons. A spinal injection facility is provided within the Leeds Primary Care Trust Musculoskeletal Service by the musculoskeletal physicians.

### Procedure

A survey was carried out within the Leeds Primary Care Musculoskeletal service. A questionnaire was designed specifically for the survey, comprising 19 questions about current practice, clinical recognition and management of patients with NC related to LSS (see additional file 1). Following a preliminary pilot with volunteer therapists, the questionnaire was modified slightly prior to conducting the full survey in November 2007.

### Data collection

Questionnaires were distributed to 54 physiotherapy staff attending a service-wide training day in November 2007. Staff were asked to complete the questionnaire without conferring with colleagues, and to return the completed questionnaire by the end of the training day.

### Data analysis

Descriptive statistics of the data were calculated using Excel. Data were predominantly categorical or descriptive, and were therefore not appropriate for inferential statistical analysis. Where open questions were used, key respondent phrases were categorised for tabular presentation and where several of the response categories were identified by a single respondent, all were included.

## Results

### Therapists' professional profiles

Fifty of 54 therapists (93%) completed and returned questionnaires. These represented a broad cross-section of staff from junior to senior physiotherapists, including seven senior therapists in 'extended physiotherapy practitioner' roles. The majority of staff (27: 54%) had been qualified for more than 10 years, and most were also very experienced in musculoskeletal work, the majority (29: 58%) having worked in the musculoskeletal field for 5 years or more (Table 1).

**Table 1:** Staff survey respondents' representation by experience

**Number of years**	**Years qualified - number of therapists**	**Years working in Musculoskeletal field - number of therapists**
**Less than 1 year**	1 (2%)	5 (10%)

**1-2 years**	4 (8%)	2 (4%)

**2-5 years**	6 (12%)	12 (24%)

**5-10 years**	11 (22%)	10 (20%)

**10 years or more**	27 (54%)	29 (58%)

**Unknown**	1 (2%)	1 (2%)

All but four respondents reported that they had received some educational training in relation to lumbar spinal stenosis or neurogenic claudication. This was provided in undergraduate training for some (12: 24%), but was more commonly accessed through post-graduate training either from external courses (33:66%), or as part of NHS within-service professional development training (30: 60%).

### Recognition & diagnosis of neurogenic claudication

Responses relating to subjective history findings in patients with NC (see Table 2) revealed that almost all respondents identified postural effects on symptoms as a significant finding, with worsening of symptoms during walking (37:74%) or standing (13:26%), and improvement or relief of symptoms with sitting (32:64%) or flexion (29:58%) of the spine (stooping). Other history findings contributing to the diagnosis of NC were reported by some, including lower limb symptoms such as cramps, paraesthesia, heaviness or sensory changes. Seven respondents (14%) identified an association with older age, whilst six (12%) mentioned a reduction in walking capacity or activity levels, and four (8%) identified a history of longstanding low back pain. A small number (3: 6%) also suggested that downhill walking might be more provocative of symptoms, although one (2%) conversely reported the expectation that uphill walking would aggravate symptoms.

**Table 2:** Subjective findings associated with NC patients by survey respondents*

**Subjective History findings**	**No. of respondents**
Worse with walking	37 (74.0%)

Eased sitting	32 (64.0%)

Eased flexion	29 (58.0%)

Worse standing	13 (26.0%)

Paraesthesia/heaviness/cramps/neural sensory changes	12 (24.0%)

Worse in spinal extension	11 (22.0%)

Age related (older patients)	7 (14.0%)

Bilateral lower limb symptoms	6 (12.0%)

Reduced walking distance/activity levels	6 (12.0%)

Longstanding LBP	4 (8.0%)

Worse downhill walking/better uphill	3 (6.0%)

Shopping trolley sign	2 (4.0%)

Worse uphill walking	1 (2.0%)

*Respondents allowed to identify more than 1 category

Whilst responses relating to clinical examination findings expected to be present in patients with NC varied more widely (see Table 3), a large proportion of respondents (29: 58%) reported that provocation of symptoms on lumbar spinal extension would increase suspicion of NC, and 21 (42%) respondents expected lumbar extension range of movement to be limited in these patients. Some (11: 22%) also reported that a stooped standing position or flattened lumbar lordosis in the patient would contribute to a clinical diagnosis of NC. Among the respondents who commented on neurological findings, there was considerable variation in expectations: seven (14%) expected neurological testing to result in abnormal findings in people with NC, while 4 (8%) expected neurological test results to be normal, and 8 (16%) reported that neurological findings may be variable in these patients.

**Table 3:** Objective findings associated with NC patients by survey respondents

**Objective clinical findings**	**No. of respondents**
Provocation of symptoms on lumbar extension	29 (58.0%)

Limited lumbar spinal extension ROM	21 (42.0%)

Stooped standing posture	8 (16.0%)

Flattened lumbar lordosis	3 (6.0%)

Normal neurological testing	4 (8.0%)

Variable neurological test findings	8 (16.0%)

Abnormal neurological testing	7 (14.0%)

Normal vascular testing	2 (4.0%)

*Respondents allowed to identify more than 1 category

A number of specific clinical tests were reportedly used to identify patients with NC by the therapists in this survey (Table 4). Some respondents reported simple clinical tests of exercise tolerance, either using a static bicycle (14:28%) or a walking test (6:12%). The majority (26:52%) suggested tests which might differentiate NC patients using mechanisms of postural alteration, such as sustained or repeated lumbar extension tests (9: 8%), a walking tolerance test in an upright position compared to a stooped position (8:16%), uphill walking compared to downhill or level walking (2: 4%), or a bicycle exercise tolerance test (with the spine in a relatively flexed position) compared to a walking exercise test (7:14%).

**Table 4:** Specific clinical tests used by respondents to diagnose NC

**Specific Clinical Tests**	**No. of respondents**
Cycle test	14 (28.0%)

Walking test	6 (12.0%)

Stooped walking test	8 (16.0%)

Cycle vs walking test	7 (14.0%)

Inclined hill walking test	2 (4.0%)

Sustained or repeated lumbar extension test	9 (18.0%)

Vascular testing	6 (12.0%)

Neurological testing	2 (4.0%)

Lasegue's sign (straight leg raise) test	3 (6.0%)

*Respondents allowed to identify more than 1 category

In addition to the clinical tests outlined above, over half of all respondents (27:54%) reported that they routinely request either MRI or X-ray investigations (or both) in patients presenting with NC (Table 5). On the other hand, 20 (40%) respondents reported that they would not normally make arrangements for any diagnostic investigations.

**Table 5:** Investigations routinely requested by therapists for patients with NC symptoms

	**Xray or MRI**	**Other tests**	**No investigations**
Number of therapists (percentage)	27 (54%) *(xray n = 21*, *MRI n = 24)*	4 (8%) *(nerve conduction n = 2*, *blood tests n = 2)*	20 (40%)

### Management of neurogenic claudication

Almost all respondents (48:96%) reported that they manage this patient group with physiotherapy treatment (Table 6). A large proportion of respondents (26:52%) also reported that they routinely refer patients with NC to be seen by one of the musculoskeletal physicians in the service and/or for a spinal injection. In addition, over half of respondents (22:44%) reported that they would routinely refer these patients for consultation with a spinal surgeon.

**Table 6:** Management routes and service referrals routinely provided or arranged by therapists for patients with NC

	**Physiotherapy treatment**	**Spinal injection clinic**	**MSK Physician referral**	**Spinal Surgeon referral**	**Pain Management service**	**Back to Fitness physio Group**	**Referral back to GP**
Number of therapists (percentage)	48 (96.0%)	26 (52.0%)	25 (50.0%)	22 (44.0%)	21 (42.0%)	8 (16.0%)	8 (16.0%)

**Figure 1:**
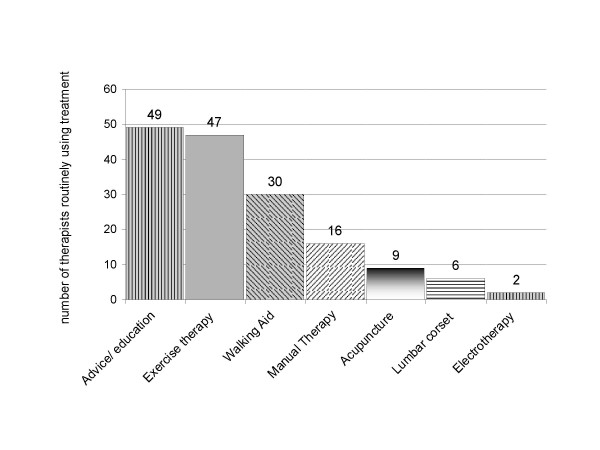
**Physiotherapy treatments routinely provided for patients with NC**.

**Figure 2:**
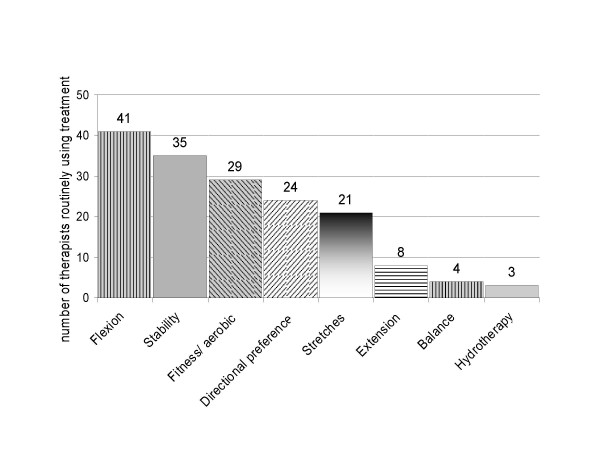
**Exercises routinely prescribed by therapists for patients with NC**.

Figures 1 and 2 describe the types of physiotherapy treatment used by respondents. Almost all respondents (47:94%) reported that they routinely include exercise therapy in addition to advice/education (49:98%) in the physiotherapy management of these patients, and the most commonly used exercises are flexion exercises (41:82%), muscle stability exercises (35:70%), and general fitness exercises (29:58%). Over half the respondents (30:60%) also reported that they routinely prescribe or suggest a walking aid for these patients.

## Discussion

The results from this survey indicate that within the Leeds primary care musculoskeletal service, NC is a recognised posture-related clinical syndrome. The survey findings show that physiotherapy treatment is used routinely in the treatment of NC. In particular, spinal flexion exercises and trunk muscle stability exercises are commonly prescribed, in addition to advice and education.

There was a high return rate for completed questionnaires, which provided a good representation of the range of physiotherapy staff grades and experience in this service. The Leeds primary care musculoskeletal service has a large proportion of highly experienced therapists. In addition, all respondents recalled receiving training at some time during their career relating to LSS or NC. The authors acknowledge that this level of experience and training may not be typical of all musculoskeletal services.

Clinical features associated with LSS and NC identified by respondents reflect those outlined in the limited literature, which suggests that radiating leg pain, exacerbation of symptoms on walking, and relief of symptoms in flexion or sitting are common findings in patients with radiologically confirmed LSS. While these symptoms of NC, along with poor balance and an age of 65 or over, have been found to be commonly associated with LSS [10,24], it is unclear whether establishing a radiological diagnosis of LSS improves outcome in the non-surgical management of patients with NC. Despite the weak correlation between clinical findings and radiological findings highlighted in the current literature [25], a high proportion of respondents indicated that they regularly refer patients with suspected NC for radiological investigations, in particular spinal x-rays or MRIs. This may be unnecessary except in cases where a surgical opinion is being sought, and recognition of the clinical syndrome of NC without expensive radiological investigations may be sufficient to guide appropriate conservative management.

Most respondents suggested the use of postural assessment in relation to symptoms to aid the differential diagnosis of NC. The need to differentiate NC from other conditions with similar presentations, in particular vascular claudication, is highlighted in the related literature [26]. Postural changes would be expected to influence symptoms of NC, but not those associated with vascular claudication. To this end, some respondents reported the use of exercise tests in different postures, such as walking compared to bicycling, or upright walking compared to stooped walking. Studies have shown that such tests may lack specificity for diagnosing pathoanatomic LSS, [24,27-29], but they may add to the clinical picture of NC.

Although conservative treatment is almost universally recommended as the first line of treatment for these patients, the few randomised trials to date have shown a superior outcome for patients with LSS undergoing surgery compared to those receiving conservative treatments [15,30-32], and many respondents reported the routine referral of patients with NC for a surgical opinion. However, a similar proportion also reported that they routinely refer for spinal injections, for which the research evidence suggests poor long-term effectiveness [33-36].

The effectiveness of physiotherapy treatments in this patient group is unclear, although evidence supports the use of physiotherapy, including exercise therapy, for chronic low back pain conditions in general [37,38]. While some research is now being directed at investigating specific treatments and exercise programmes for defined subgroups of low back pain [39-41], very little research has focussed on the effects of exercises in older low back pain patients or patients with the specific posture-related symptoms of NC.

Despite the lack of research evidence, it is almost universally recommended in the literature that conservative treatments, including physiotherapy, are used as the first line of treatment for these patients [2,26,42,43]. The fact that almost all respondents in this survey routinely treat NC patients with physiotherapy reflects this recommendation. It is interesting, given the paucity of research evidence to guide treatment choice, that the types of physiotherapy treatments routinely employed by the therapists in this survey are fairly similar. In addition to advice and education, most therapists routinely prescribe an exercise programme. The choice of exercise treatments generally reflects approaches suggested in the literature, based on the theoretical benefits potentially resulting from minimising lumbar extension positions. These include exercises which encourage flexed postures through flexion-based and trunk stabilising exercises [44-47]. Evidence for the efficacy of such treatments, however, is still lacking.

## Conclusion

This survey shows that musculoskeletal physiotherapy clinicians recognise a clear clinical syndrome of NC, based on the findings of posture-dependent symptoms. Whilst a high proportion of therapists in this survey reported the regular use of diagnostic imaging for patients with symptoms of NC, it is not known whether establishing a radiological diagnosis of LSS improves the management of these patients.

Non-surgical management is recommended for patients with NC and LSS in the first instance, but there are no evidence-based guidelines to inform the choice of conservative treatments. Most therapists in this survey reported that they routinely provide physiotherapy treatments which encourage flexion postures and movements; an approach which, despite a lack of evidence, is commonly recommended in the literature.

## Competing interests

The authors declare that they have no competing interests.

## Authors' contributions

CMC carried out the data collection, and participated in the design of the study, analysis of the data, and preparation of the manuscript. ACR, HAB and PGC participated in the study design, analysis of the data and manuscript preparation. All authors read and approved the final manuscript.

## Pre-publication history

The pre-publication history for this paper can be accessed here:



## Supplementary Material

Additional file 1 **Questionnaire survey form**. Questionnaire used for survey of current practice in the management of neurogenic claudication related to degenerative lumbar spinal stenosis in Leeds Primary Care Musculoskeletal Service. Click here for file

## References

[B1] Goh KJ, Khalifa W, Anslow P, Cadoux HT, Donaghy M (2004). The clinical syndrome associated with lumbar spinal stenosis. European neurology.

[B2] Agency for Healthcare Research and Quality (2001). Treatment of degenerative lumbar spinal stenosis: summary. Evidence report/technology assessment number 32. AHRQ publication no 01-E047.

[B3] Lurie JD, Birkmeyer NJ, Weinstein JN (2003). Rates of advanced spinal imaging and spine surgery. Spine.

[B4] Turner JA, Ersek M, Herron L, Deyo R (1992). Surgery for lumbar spinal stenosis. Attempted meta-analysis of the literature. Spine.

[B5] Porter RW (1996). Spinal stenosis and neurogenic claudication. Spine.

[B6] Bal S, Celiker R, Palaoglu S, Cila A (2006). F wave studies of neurogenic intermittent claudication in lumbar spinal stenosis. American journal of physical medicine & rehabilitation/Association of Academic Physiatrists.

[B7] Inufusa A, An HS, Lim TH, Hasegawa T, Haughton VM, Nowicki BH (1996). Anatomic changes of the spinal canal and intervertebral foramen associated with flexion-extension movement. Spine.

[B8] Schonstrom N, Lindahl S, Willen J, Hansson T (1989). Dynamic changes in the dimensions of the lumbar spinal canal: an experimental study in vitro. J Orthop Res.

[B9] Garfin SR, Rauschning W (2001). Spinal stenosis. Instructional course lectures.

[B10] Katz JN, Dalgas M, Stucki G, Katz NP, Bayley J, Fossel AH, Chang LC, Lipson SJ (1995). Degenerative lumbar spinal stenosis. Diagnostic value of the history and physical examination. Arthritis and rheumatism.

[B11] Iversen MD, Katz JN (2001). Examination findings and self-reported walking capacity in patients with lumbar spinal stenosis. Physical therapy.

[B12] de Graaf I, Prak A, Bierma-Zeinstra S, Thomas S, Peul W, Koes B (2006). Diagnosis of lumbar spinal stenosis: a systematic review of the accuracy of diagnostic tests. Spine.

[B13] Haig AJ, Tong HC, Yamakawa KS, Parres C, Quint DJ, Chiodo A, Miner JA, Phalke VC, Hoff JT, Geisser ME (2006). Predictors of pain and function in persons with spinal stenosis, low back pain, and no back pain. Spine.

[B14] Atlas SJ, Delitto A (2006). Spinal stenosis: surgical versus nonsurgical treatment. Clin Orthop Relat Res.

[B15] Amundsen T, Weber H, Nordal HJ, Magnaes B, Abdelnoor M, Lilleas F (2000). Lumbar spinal stenosis: conservative or surgical management?: A prospective 10-year study. Spine.

[B16] Simotas AC, Dorey FJ, Hansraj KK, Cammisa F (2000). Nonoperative treatment for lumbar spinal stenosis. Clinical and outcome results and a 3-year survivorship analysis. Spine.

[B17] Houedakor J, Cabre P, Pascal-Moussellard H, Gallien P, Rene-Corail P, Smadja D (2003). [Rehabilitation treatment in lumbar canal stenosis. Intermediate results of a prospective study (Telemar)]. Ann Readapt Med Phys.

[B18] Zeifang F, Abel R, Schiltenwolf M (2003). Possible conservative treatment methods for patients with spinal claudication. Der Orthopade.

[B19] Simotas AC (2001). Nonoperative treatment for lumbar spinal stenosis. Clin Orthop Relat Res.

[B20] Fritz JM, Erhard RE, Vignovic M (1997). A nonsurgical treatment approach for patients with lumbar spinal stenosis. Physical therapy.

[B21] DuPriest CM (1993). Nonoperative management of lumbar spinal stenosis. J Manipulative Physiol Ther.

[B22] Whitman JM, Flynn TW, Fritz JM (2003). Nonsurgical management of patients with lumbar spinal stenosis: a literature review and a case series of three patients managed with physical therapy. Physical medicine and rehabilitation clinics of North America.

[B23] Whitman JM, Flynn TW, Childs JD, Wainner RS, Gill HE, Ryder MG, Garber MB, Bennett AC, Fritz JM (2006). A comparison between two physical therapy treatment programs for patients with lumbar spinal stenosis: a randomized clinical trial. Spine.

[B24] Fritz JM, Erhard RE, Delitto A, Welch WC, Nowakowski PE (1997). Preliminary results of the use of a two-stage treadmill test as a clinical diagnostic tool in the differential diagnosis of lumbar spinal stenosis. Journal of spinal disorders.

[B25] Haig AJ, Geisser ME, Tong HC, Yamakawa KS, Quint DJ, Hoff JT, Chiodo A, Miner JA, Phalke VV (2007). Electromyographic and magnetic resonance imaging to predict lumbar stenosis, low-back pain, and no back symptoms. The Journal of bone and joint surgery.

[B26] Fritz JM, Delitto A, Welch WC, Erhard RE (1998). Lumbar spinal stenosis: a review of current concepts in evaluation, management, and outcome measurements. Archives of physical medicine and rehabilitation.

[B27] Goldman SM, Barice EJ, Schneider WR, Hennekens CH (2008). Lumbar spinal stenosis: can positional therapy alleviate pain?. The Journal of family practice.

[B28] Dong G, Porter RW (1989). Walking and cycling tests in neurogenic and intermittent claudication. Spine.

[B29] Dyck P (1979). The stoop-test in lumbar entrapment radiculopathy. Spine.

[B30] Mariconda M, Fava R, Gatto A, Longo C, Milano C (2002). Unilateral laminectomy for bilateral decompression of lumbar spinal stenosis: a prospective comparative study with conservatively treated patients. Journal of spinal disorders & techniques.

[B31] Malmivaara A, Slatis P, Heliovaara M, Sainio P, Kinnunen H, Kankare J, Dalin-Hirvonen N, Seitsalo S, Herno A, Kortekangas P (2007). Surgical or nonoperative treatment for lumbar spinal stenosis? A randomized controlled trial. Spine.

[B32] Weinstein JN, Tosteson TD, Lurie JD, Tosteson AN, Blood E, Hanscom B, Herkowitz H, Cammisa F, Albert T, Boden SD (2008). Surgical versus nonsurgical therapy for lumbar spinal stenosis. N Engl J Med.

[B33] Fukusaki M, Kobayashi I, Hara T, Sumikawa K (1998). Symptoms of spinal stenosis do not improve after epidural steroid injection. The Clinical journal of pain.

[B34] Cuckler JM, Bernini PA, Wiesel SW, Booth RE, Rothman RH, Pickens GT (1985). The use of epidural steroids in the treatment of lumbar radicular pain. A prospective, randomized, double-blind study. The Journal of bone and joint surgery.

[B35] Ng LC, Sell P (2004). Outcomes of a prospective cohort study on peri-radicular infiltration for radicular pain in patients with lumbar disc herniation and spinal stenosis. Eur Spine J.

[B36] Ng L, Chaudhary N, Sell P (2005). The efficacy of corticosteroids in periradicular infiltration for chronic radicular pain: a randomized, double-blind, controlled trial. Spine.

[B37] Hayden JA, van Tulder MW, Tomlinson G (2005). Systematic review: strategies for using exercise therapy to improve outcomes in chronic low back pain. Annals of internal medicine.

[B38] Airaksinen O, Brox JI, Cedraschi C, Hildebrandt J, Klaber-Moffett J, Kovacs F, Mannion AF, Reis S, Staal JB, Ursin H (2006). Chapter 4. European guidelines for the management of chronic nonspecific low back pain. Eur Spine J.

[B39] O'Sullivan P (2005). Diagnosis and classification of chronic low back pain disorders: maladaptive movement and motor control impairments as underlying mechanism. Manual therapy.

[B40] Childs JD, Fritz JM, Flynn TW, Irrgang JJ, Johnson KK, Majkowski GR, Delitto A (2004). A clinical prediction rule to identify patients with low back pain most likely to benefit from spinal manipulation: a validation study. Annals of internal medicine.

[B41] Long A, Donelson R, Fung T (2004). Does it matter which exercise? A randomized control trial of exercise for low back pain. Spine (Phila Pa 1976).

[B42] Atlas SJ, Keller RB, Robson D, Deyo RA, Singer DE (2000). Surgical and nonsurgical management of lumbar spinal stenosis: four-year outcomes from the maine lumbar spine study. Spine.

[B43] Yuan PS, Albert TJ (2004). Nonsurgical and surgical management of lumbar spinal stenosis. Journal of Bone and Joint Surgery-American Volume.

[B44] Bodack MP, Monteiro M (2001). Therapeutic exercise in the treatment of patients with lumbar spinal stenosis. Clin Orthop Relat Res.

[B45] Rittenberg JD, Ross AE (2003). Functional rehabilitation for degenerative lumbar spinal stenosis. Physical medicine and rehabilitation clinics of North America.

[B46] Duarte M (2002). Therapeutic exercise as a treatment for lumbar spinal stenosis. Journal of American Chiropractic Association.

[B47] Nagler W, Hausen HS (1998). Conservative management of lumbar spinal stenosis. Identifying patients likely to do well without surgery. Postgraduate medicine.

