# Supplementary material for: Assessment and management of neurogenic claudication associated with lumbar spinal stenosis in a UK primary care musculoskeletal service: a survey of current practice among physiotherapists
Source: BMC Musculoskelet Disord. 2009 Oct 1;10:121. doi: 10.1186/1471-2474-10-121 (PMC2762954; doi:10.1186/1471-2474-10-121)
Supplement: Additional file 1 — Questionnaire survey form. Questionnaire used for survey of current practice in the management of neurogenic claudication related to degenerative lumbar spinal stenosis in Leeds Primary Care Musculoskeletal Service. [file 1471-2474-10-121-S1.DOC]

**CURRENT PRACTICE IN THE MANAGEMENT OF NEUROGENIC CLAUDICATION RELATED TO DEGENERATIVE LUMBAR SPINAL STENOSIS IN LEEDS PRIMARY CARE MUSCULOSKELETAL SERVICE**

This questionnaire is intended for Physiotherapy/Osteopathic/Chiropractic staff working in the Leeds Primary Care Musculoskeletal Service.

There are no right or wrong answers and you do not need to put your name on this questionnaire. In order to provide a more accurate view of current practice in the management of patients with lumbar spinal stenosis, please complete the questionnaire without conferring with colleagues or referring to literature or other material.

The data will be used to evaluate current practice in the management of patients with degenerative lumbar spinal stenosis, and may be used for audit purposes.

**THANK YOU!**

**The following questions are about you and your work**

**1. What is your profession?** (eg physiotherapist, osteopath)

**2. What is your current job title?** (eg physiotherapist, ESP, superintendent physio)

**3. Which area of Leeds do you work in?**

❑ West

❑ North West

❑ East

❑ North East

❑ South

**4. How long have you been qualified?**

❑ Less than 1 year

❑ 1-2 yrs

❑ 2-5 yrs

❑ 5-10 yrs

❑ More than 10 yrs

**5. How long have you worked in the musculoskeletal field?**

❑ Less than 1 year

❑ 1-2 yrs

❑ 2-5 yrs

❑ 5-10 yrs

❑ More than 10 yrs

**6. How many NEW spinal patients do you assess in an average week?**

❑ 0

❑ 1-5

❑ 6-10

❑ more than 10

**Neurogenic claudication is the classical clinical presentation of patients with degenerative lumbar spinal stenosis. The following questions are about your experience in assessing patients with neurogenic claudication/ lumbar spinal stenosis**

**7. How many patients have you assessed who you have suspected of having neurogenic claudication/ spinal stenosis in the last 6 months?**

❑ 0

❑ 1-5

❑ 6-10

❑ more than 10

**8. What main subjective findings from the patient history would lead you to suspect neurogenic claudication/ degenerative lumbar spinal stenosis?**

|  |
| --- |
|  |
|  |
|  |
|  |
|  |

**9. What main objective findings on clinical assessment of the patient would lead you to suspect degenerative lumbar spinal stenosis?**

|  |
| --- |
|  |
|  |
|  |
|  |
|  |

**10. Are there any specific clinical tests that you find helpful in assessing patients who you think might have lumbar spinal stenosis?**

|  |
| --- |
|  |
|  |
|  |
|  |
|  |

**The following questions relate to the management of patients with neurogenic claudication/ degenerative lumbar spinal stenosis**

**11. Would you routinely organise investigations for patients who you suspect to have neurogenic claudication/ degenerative lumbar spinal stenosis?** (please tick, even if you do this indirectly through an ESP/MSK Doctor)

❑ Xray

❑ MRI

❑ CT

❑ Ultrasound

❑ Nerve conduction studies

❑ Myelography

❑ Blood tests

❑ None of the above

**12. Would you routinely treat patients with neurogenic claudication/ degenerative lumbar spinal stenosis with physiotherapy?**

❑ Yes

**❑** No

**If Yes, which of the following would you routinely include?**

|  | Tick any that apply | Further details of treatments given and what would influence your choice of treatment |
| --- | --- | --- |
| Advice on self management |  |  |
| Manual therapy |  |  |
| Exercise therapy |  |  |
| Acupuncture treatment |  |  |
| Electrotherapy treatment |  |  |
| Walking aids |  |  |
| Spinal corset |  |  |
| Other – please give details |  |  |

**13. Would you routinely use any other management options for these patients?**

|  | Tick any that apply | Further details of treatments given and what would influence your choice of treatment |
| --- | --- | --- |
| Referral back to GP |  |  |
| Referral to spinal injection clinic |  |  |
| Referral to pain management clinic |  |  |
| Referral to 'back to fitness' class |  |  |
| Referral to musculoskeletal doctor |  |  |
| Referral to spinal surgeon |  |  |
| Other – please give details |  |  |

14. Are there any particular factors which would influence your choice of management?

|  |
| --- |
|  |
|  |
|  |

**15. If you would normally include exercise therapy in your management of patients with degenerative lumbar spinal stenosis, what kind of exercises would you routinely use?**

❑ Repeated movements (directional preference exercises)

❑ Flexion-based exercises

❑ Extension-based exercises

❑ Stability exercises

❑ General fitness/aerobic/cardiovascular exercises

❑ Stretches

❑ Balance/proprioception exercises

❑ Hydrotherapy

❑ other – please give details

|  |
| --- |
|  |
|  |

**The following questions are about your training in relation to lumbar spinal stenosis**

**16. Did you receive any training or education about lumbar spinal stenosis as an**

**undergraduate?**

❑ Yes

❑ No

❑ Not that I can remember

**17. Have you attended any courses relating to the lumbar spine since qualifying?**

❑ None

❑ McKenzie

❑ MACP

❑ Society of Orthopaedic Medicine

❑ Kinetic Control

❑ Msc course/module

❑ Conference

❑ Other – please give details

|  |
| --- |
|  |

**18. If you *have* attended any postgraduate lumbar spine courses, did the course(s) include any training/education in relation to lumbar spinal stenosis?**

    Yes No Not sure/don't remember

McKenzie ❑ ❑ ❑

MACP ❑ ❑ ❑  

Society of Orthopaedic Medicine ❑ ❑ ❑

Kinetic Control ❑ ❑ ❑

Msc course/module ❑ ❑ ❑

Conference ❑ ❑ ❑

Other ❑ ❑ ❑

|  |
| --- |
|  |

**19. Have you attended any training at work (eg in-service training) relating to lumbar spinal stenosis ?**

❑ Yes

❑ No

***THANK YOU!***
